# Eupafolin and Ethyl Acetate Fraction of *Kalanchoe gracilis* Stem Extract Show Potent Antiviral Activities against Enterovirus 71 and Coxsackievirus A16

**DOI:** 10.1155/2013/591354

**Published:** 2013-09-02

**Authors:** Ching-Ying Wang, Shun-Chueh Huang, Zhen-Rung Lai, Yu-Ling Ho, Yu-Jen Jou, Szu-Hao Kung, Yongjun Zhang, Yuan-Shiun Chang, Cheng-Wen Lin

**Affiliations:** ^1^School of Chinese Pharmaceutical Sciences and Chinese Medicine Resources, China Medical University, Taichung 404, Taiwan; ^2^Department of Medical Laboratory Science and Biotechnology, China Medical University, Taichung 404, Taiwan; ^3^School of Pharmacy, China Medical University, Taichung 404, Taiwan; ^4^Department of Nursing, Hungkuang University, Taichung 433, Taiwan; ^5^Institute of Biochemistry, National Chung Hsing University, Taichung 402, Taiwan; ^6^Department of Biotechnology and Laboratory Science in Medicine, National Yang Ming University, Taipei 112, Taiwan; ^7^Fujian Center for Disease Control and Prevention, Fuzhou, Fujian 350001, China; ^8^Department of Biotechnology, Asia University, Wufeng, Taichung 413, Taiwan

## Abstract

Enterovirus 71 (EV71) and coxsackievirus A16 (CoxA16) are main pathogens of hand-foot-and-mouth disease, occasionally causing aseptic meningitis and encephalitis in tropical and subtropical regions. *Kalanchoe gracilis*, *Da-Huan-Hun*, is a Chinese folk medicine for treating pain and inflammation, exhibiting antioxidant and anti-inflammatory activities. Our prior report (2012) cited *K. gracilis* leaf extract as moderately active against EV71 and CoxA16. This study further rates antienteroviral potential of *K. gracilis* stem (KGS) extract to identify potent antiviral fractions and components. The extract moderately inhibits viral cytopathicity and virus yield, as well as *in vitro* replication of EV71 (IC_50_ = 75.18 **μ**g/mL) and CoxA16 (IC_50_ = 81.41 **μ**g/mL). Ethyl acetate (EA) fraction of KGS extract showed greater antiviral activity than that of *n*-butanol or aqueous fraction: IC_50_ values of 4.21 **μ**g/mL against EV71 and 9.08 **μ**g/mL against CoxA16. HPLC analysis, UV-Vis absorption spectroscopy, and plaque reduction assay indicate that eupafolin is a vital component of EA fraction showing potent activity against EV71 (IC_50_ = 1.39 **μ**M) and CoxA16 (IC_50_ = 5.24 **μ**M). Eupafolin specifically lessened virus-induced upregulation of IL-6 and RANTES by inhibiting virus-induced ERK1/2, AP-1, and STAT3 signals. Anti-enteroviral potency of KGS EA fraction and eupafolin shows the clinical potential against EV71 and CoxA16 infection.

## 1. Introduction

Enteroviruses (EVs) like polio, enterovirus 71 (EV71), and coxsackieviruses A (CoxA) belong to the Picornaviridae family, causing severe manifestations: for example, hand, foot, and mouth disease (HFMD); meningitis; encephalitis; flaccid paralysis; myocarditis [1–3]. EV consists of single-strand, positive-sense RNA approximately 7.4 kb in size and a nonenveloped capsid (27–30 nm in diameter). Viral genome has a long open reading frame encoding polyprotein cleaved to form four structural proteins (VP1, VP2, VP3, and VP4) and seven nonstructural proteins (2A–C, 3A–D) by proteases 2A^pro^ and 3C^pro^ [2, 4]. EV71 and CoxA16 are two major causative agents of HFMD in children with severe brain stem encephalitis [5]. Several EV71 outbreaks with severe or even fatal cases occurred in Malaysia in 1997, Taiwan in 1998, Japan in 2000, Vietnam in 2005, and Singapore in 2008. Recent mixed infection of EV71 and CoxA16 in HFMD cases appeared in China and India during 2009-2010 [5–7]. No vaccine or antiviral agent for EV infection is currently available.

*Kalanchoe gracilis* (L.), a.k.a. *Da*-*Huan*-*Hun*, is a Chinese folk medicine in Taiwan, commonly used to alleviate pain, fever, inflammation, and injuries [8–10]. Its bioactive compounds include coumarin, bufadienolides, flavonoids (quercetin, kaempferol, teolin, quercitrin, and eupafolin), and glycosidic derivatives of eupafolin demonstrating antioxidant, anti-inflammatory, and/or antiproliferative activities [11–13].* K. gracilis* leaf extract with ferulic acid, quercetin, and kaempferol shows moderately antiviral activity against CoxA16 and EV71 *in vitro* and *in vivo* [14]. *K. gracilis* stem extract exhibits potent analgesic and anti-inflammatory activities in acetic acid-induced writhing responses, elevating superoxide dismutase activities in the liver and reducing TNF-alpha levels of inflamed animal model tissues [9]. Methanolic extract of *K. gracilis* stem has potent antioxidant, anti-inflammation, and antiproliferative activities *in vitro* [10]. Eupafolin (6-methoxyluteolin), identified in the stem of *K. gracilis*, significantly reduces nitric oxide (NO) production and expressions of inducible nitric oxide synthase (iNOS) and cyclooxygenase-2 (COX-2) in LPS-treated RAW264.7 macrophage cells [10]. Eupafolin is the crucial and bioactive component of antioxidant, anti-inflammatory, and antiproliferative activities in many medicinal herbs: for example, *Artemisia princeps*, *Eupatorium perfoliatum* L., and *Gaillardia aristata* Pursh [11–13, 15].

This study investigated antiviral effects of *K. gracilis *stem (KGS) extract, ethyl acetate (EA), and *n*-butanol (BuOH) fractions against EV71 and CoxA16, ferreting out potential antiviral compounds of *K. gracilis *stem extract. EA fraction, better than BuOH fraction, effectively inhibited virus-induced cytopathicity and viral replication *in vitro*. Eupafolin, rich in EA fraction, showed a potent antiviral activity, with IC_50_ values of less than 10 *μ*M, inhibiting production of proinflammatory cytokines via suppressing ERK1/2 and AP-1 mediated signaling pathways.

## 2. Materials and Methods

### 2.1. Fractionation of *K. gracilis* Stem (KGS) Extract

*K. gracilis* was collected from farmlands and gardens in Chiayi County, as detailed in our prior report [14]; its stem juice filtered by Whatman No. 1 paper, and then lyophilized in an IWAKI FDR-50P freeze dryer. Powder of stem extract was stored in sterile bottles at −20°C, dissolved in distilled deionised water, then partitioned with ethyl acetate (*V/V* = 1/1). Water fraction was mixed with *n*-butanol (*V/V* = 1/1), with ethyl acetate (EA), *n*-butanol (BuOH), and aqueous (H_2_O) fractions evaporated under reduced pressure by BUCHI Rotavapor R-114.

### 2.2. Viruses and Cells

EV71 and CoxA16 strains were amplified in RD cells grown in Dulbecco's Modified Eagle's Medium with 10% fetal bovine serum (FBS) at 37°C, 5% CO_2_, as detailed in our prior report [14]. HeLa-G2AwtR cells were maintained in Modified Eagle's Medium with 10% FBS and 20 *μ*g/mL zeocin, expressing FRET probe as well as fusion protein of red fluorescent protein (DsRed)-2Apro cleavage motif-green fluorescent protein (GFP) [16].

### 2.3. Cell Viability Assay

In all, 3 × 10^4^ RD cells were added to each well of 96-well plates, cultured at 37°C, 5% CO_2_ overnight, then quintuplicate treated with KGS extract, indicated fraction (EA, BuOH, or H_2_O), eupafolin or caffeic acid for an additional 48-hour incubation. Cell survival rate was calculated as ratio of optical density (OD)_570 nm_ − OD_630 nm_(OD_570−630_) of treated cells to OD_570−630_ of mock cell using MTT assay [14]. Data showed means ± SD from three independent experiments; 50% cytotoxic concentration (CC_50_) yielding 50% toxic effect was determined via computer program (provided by John Spouge, National Center for Biotechnology Information, National Institutes of Health).

### 2.4. Cytopathic Effect (CPE) Reduction and Virus Yield

RD cells cultured in 6-well plates were infected with EV71 or CoxA16 at multiplicity of infection (MOI) 0.1 in the presence or absence of various amounts of KGS extract, indicated fraction, eupafolin or caffeic acid for 24 or 48 hours. Cellular morphology was observed and photographed under microscope. To quantify virus yield, cultured supernatants from each treated/infected cells were harvested 12, 24, 36, or 48 hours postinfection, then counted by real-time RT-PCR with VP1-specific primers, as described in our prior report [14]. The Ct value for viral yield in cultured supernatant was monitored by ABI PRISM 7000 sequence detection system (Applied Biosystems), delta Ct value calculated by subtracting Ct value for viral yield in cultured supernatant of infected cells with indicated treatment from that of cultured media of infected cells without treatment.

### 2.5. Plaque Reduction Assay

Monolayer of RD cells cultured in 6-well plates was infected with EV71 or CoxA16 (50 pfu per well) in the presence or absence of KGS extract (1, 10, 50, 100, 150 *μ*g/mL), indicated fraction (0.5, 1, 5, 10, 20 *μ*g/mL), and eupafolin or caffeic acid (0.1, 1, 5, 10, 20, 50 *μ*g/mL) for 1 h in a 37°C, 5% CO_2_ incubator. Medium containing 3% agarose (2 mL per well) was used to cover cell monolayer. After 2-day incubation at 37°C in a CO_2_ incubator, cell monolayer was stained with 0.1% Crystal Violet and plaque number further counted. Fifty percent (50%) inhibitory concentration (IC_50_) reducing number of plaques by 50% was calculated by computer program, selectivity index (SI) derived by ratio of CC_50_ to IC_50_. Data presented are mean values of three independent experiments; error bars represent standard deviations.

### 2.6. Cell Cycle Analysis Using Flow Cytometry

A total of 2 × 10^5^ RD cells were infected with EV71 or CoxA16 at MOI of 0.5 in the presence and absence of 10 *μ*g/mL EA or BuOH fraction, then collected 36 h postinfection, and washed with PBS buffer. After centrifuging at 8000 rpm for 3 min, cell pellet dissolved with 100 *μ*L PBS buffer was incubated 1 mL of 70% alcohol in −20°C overnight and washed twice with PBS buffer for propidium iodide (PI) staining (Biolegend). After 30 min incubation with PI/RNAase solution, over 10,000 stained cells were analyzed by BD FACSAria (Becton Dickinson) with excitation at 488 nm and emission at 633 nm.

### 2.7. Fingerprint Analysis by HPLC

Marker compounds of *K. gracilis* (ferulic acid, quercetin, kameperfol, eupafolin, and caffeic acid) were obtained from ChromaDex, Inc. and Sigma-Aldrich Chemical Co. Fingerprint profiles of KGS EA and BuOH fractions were analyzed and compared with retention time of marker compounds, using Waters 2695 Separations Module in the HPLC instrument (Waters 2695 Separations Module, Waters 2996 Photodiode Array Detector, Atlantis dC18 5 *μ*m 4.6 × 250 mm column). Mobile phase was 0.2% formic acid and acetonitrile (70 : 30), chromatographic separation set at 1.0 mL/min flow rate, and elution peaks detected at 345 nm with a 2996 PDA detector.

### 2.8. Virucidal and Virus Attachment Assays

For virucidal assay, EV71 or CoxA16 (10^6^ pfu) was incubated with EA, BuOH fraction (10, 100 *μ*g/mL), or eupafolin (1, 10 *μ*g/mL) for 1 h at 4°C. Mixture was further diluted by 100- and 1000-fold, infectious activity performed by plaque assay. For virus attachment assay, EV71 or CoxA16 (50 pfu) was added to the RD cell monolayer in 6-well plates, concomitant with EA, BuOH fraction, or eupafolin. After 1 h incubation at 4°C, cell monolayer was washed twice with PBS, then overlaid with 2 mL of culture medium containing 3% agarose for 2 days at 37°C in a CO_2_ incubator. Virus attachment activity was calculated as residual plaques after staining with 0.1% crystal violet solution.

### 2.9. 2A Protease Activity Assay Using FRET

HeLa-G2AwtR cells expressed fusion substrates as FRET probes containing 2A protease specific cleavage peptides at the middle region. Cells seeded into the 6-well tissue culture plates were infected with EV71 or CoxA16 at MOI of 1 in the presence or absence of 10 *μ*g/mL of EA or BuOH fraction and 1 *μ*g/mL eupafolin. Two days postinfection, cells were harvested, and then fluorescent intensity of the FRET probes in lysates was determined by fluorescent-plate reader with excitation wavelength at 390/20 nm (for GFP^2^) and emission wavelength at 590/14 nm (for DsRed2), in which DsRed2 was excited by emission wavelength of GFP^2^ at 510/10 nm. EV71 and CoxA16 infection substantially abrogated FRET; treatment with 2A protease inhibitors will restore FRET.

### 2.10. Quantification of RANTES and IL-6 Gene Expression Using Real-Time RT-PCR

Total RNA was isolated from virus-infected RD cells treated with interferon- (IFN-)*α*, EA fraction, or eupafolin by purification kit (PureLink. TM. Micro-to-Midi. TM. total. RNA purification system, Invitrogen) used for cDNA synthesis with oligo dT primer and SuperScript III reverse transcriptase kit (Invitrogen). To gauge mRNA expression in response to EA fraction or eupafolin treatment and/or virus infection, two-step RT-PCR with SYBR Green I was used. Oligonucleotide primer pairs included 5′-TCCCCATATTCCTCGGAC-3′ and 5′-GATGTACTCCCGAACCCA-3′ for human RANTES, 5′-GATGGATGCTTCCAATCTGGAT-3′ and 5′-AGTTCTCCATAGAGAACAACATA-3′ for IL-6, and 5′-AGCCACATCGCTCAGACAC-3′ and 5′-GCCCCAATACGACCAAATCC-3′ for GAPDH. Real-time PCR reaction was carried out using ABI PRISM 7700 sequence detection system, as described in prior study [14]. Relative mRNA expression levels of indicated were normalized by housekeeping gene GAPDH.

### 2.11. Western Blot Analysis

Lysates from virus-infected RD cells treated with interferon-alpha, EA fraction, or eupafolin were dissolved in SDS-PAGE sample buffer with 2-mercaptoethanol, boiled for 10 min, then applied to run 8% SDS-PAGE gels. After transfer, resulting blots were blocked with 5% skim milk in TBST, incubated with antiphospho-STAT3 (Tyr705), antiphospho-ERK1/2, antiphospho-p38 MAPK, antiphospho-p65 (NF-*κ*B), antiphospho-c-Jun, or anti-*β*-actin antibodies (Cell Signaling Technology), respectively. Immunoreactive bands were developed by horseradish peroxidase-conjugated secondary antibodies and enhanced chemiluminescent substrates (Amersham Pharmacia Biotech).

### 2.12. *In Vivo* Anti-EV71 Assay

The 1-day-old suckling mice were intraperitoneally infected with 1.7 × 10^7^ pfu EV71 then intraperitoneally injected with *K. gracilis* stem extract (5 mg/kg) once on days 1, 3, 5, and 7. Three mice from each group were sacrificed on days 2, 4, 6, and 8; their intestine samples were collected for detection of virus loads using real-time RT PCR, described as in Section 2.4.

### 2.13. Statistical Analysis

Data from three independent experiments were represented as mean ± standard deviation (SD) and statistically analyzed, using SPSS program (version 10.1, SPSS Inc., IL) via one-way ANOVA analysis by Scheffe's test.

## 3. Results

### 3.1. Antiviral Activity of KGS Extract against EV71 and CoxA16

**Figure 1:**
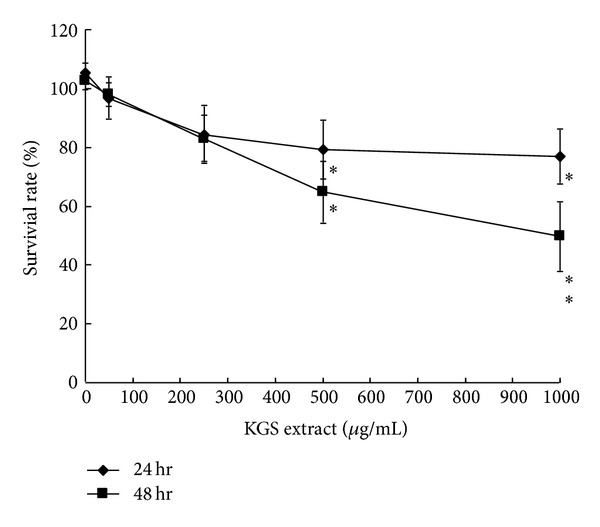
Survival rate of RD cells treated with KGS extract. RD cells cultured on 96-well plates were treated with indicated concentrations of KGS extract, then incubated for 24 or 48 hours, followed by MTT assay. Survival rates of cells were calculated as the ratio of OD_570−630 nm_ of treated cells to OD_570−630 nm_ of untreated cells. *P value < 0.05 by Scheffe's test.

**Figure 2:**
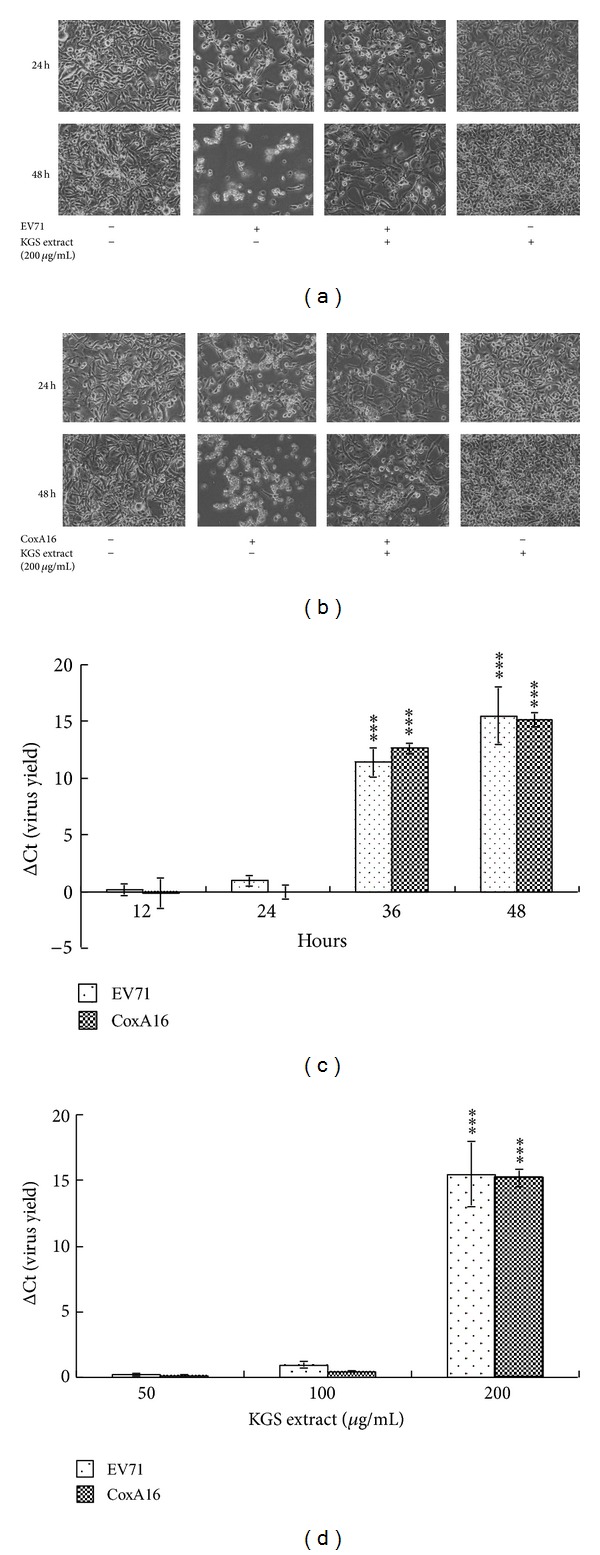
Inhibitory effects of KGS extract on viral cytopathicity and yield in RD cells. EV71 or CoxA16 at MOI of 0.1 mixed with indicated KGS extract concentration was immediately added to RD cell culture. Virus-induced cytopathic effect was photographed 36 h postinfection by reverse-phase microscopy ((a), (b)). Virus yield in each cultured supernatant was measured by real-time RT-PCR in time- (c) or concentration- (d) dependent manner. Delta Ct value was calculated by subtracting Ct value for viral load in cultured supernatant of KGS extract-treated infected cells from Ct value for viral load in cultured supernatant of infected cells without treatment. ***P value < 0.001 by Scheffe's test.

**Figure 3:**
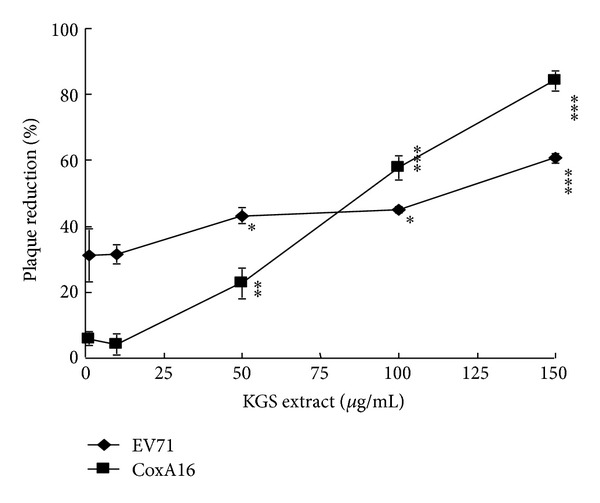
Plaque reduction by KGS extract. Monolayer of RD cells on six-well plates infected with EV71 or CVA16 at a titer of 100 pfu was treated simultaneously with indicated KGS extract concentrations. After 1 h incubation, monolayer was covered with 2 mL of agarose overlay medium and incubated for 2 days at 37°C in a CO_2_ incubator. Finally, plaques were counted after crystal violet staining.

KGS extract has a CC_50_ value of 1622 *μ*g/mL to RD cells 48 h posttreatment, showing low cytotoxicity (Figure 1, Table 1). Subsequently, antienterovirus ability of KGS extract was rated with cytopathicity, virus yield, and plaque reduction assays. KGS extract (200 *μ*g/mL) reduced cytopathicity of RD cells induced by EV71 and CoxA16 (Figures 2(a)-2(b)). Virus titer assay of cultured supernatants using real-time RT PCR assay indicated KGS extract* in vitro* significantly inhibiting EV71 and CoxA16 replication in both time- and concentration-dependent manner (Figures 2(c)-2(d)). For determining potency and selectivity, plaque reduction assay was further performed (Figure 3), revealing IC_50_ values of KGS extract as 75.18 *μ*g/mL and 81.41 *μ*g/mL for EV71 and CoxA16, respectively. Meanwhile, selectivity index (SI) of KGS extract was approximately 20 (Table 1). Antiviral activity of KGS extract in 1-day suckling mice was also examined in viral loads by real-time PCR (see Supplementary Material, Supplemental Table 1, available online at http://dx.doi.org/10.1155/2013/591354). In mock group, EV71 in intestine samples was detectable 2, 4, and 6 days postintraperitoneal inoculation. By contrast, intraperitoneal treatment with KGS extract resulted in the decrease of EV71 loads compared to the mock group 2 days postinoculation, as not detectable 4, and 6 days postinoculation. Results demonstrate KGS extract consisting of active anti-EV71 and CoxA16 components *in vitro* and* in vivo*.

### 3.2. Functional Fractions of KGS Extract against EV71 and CoxA16

**Figure 4:**
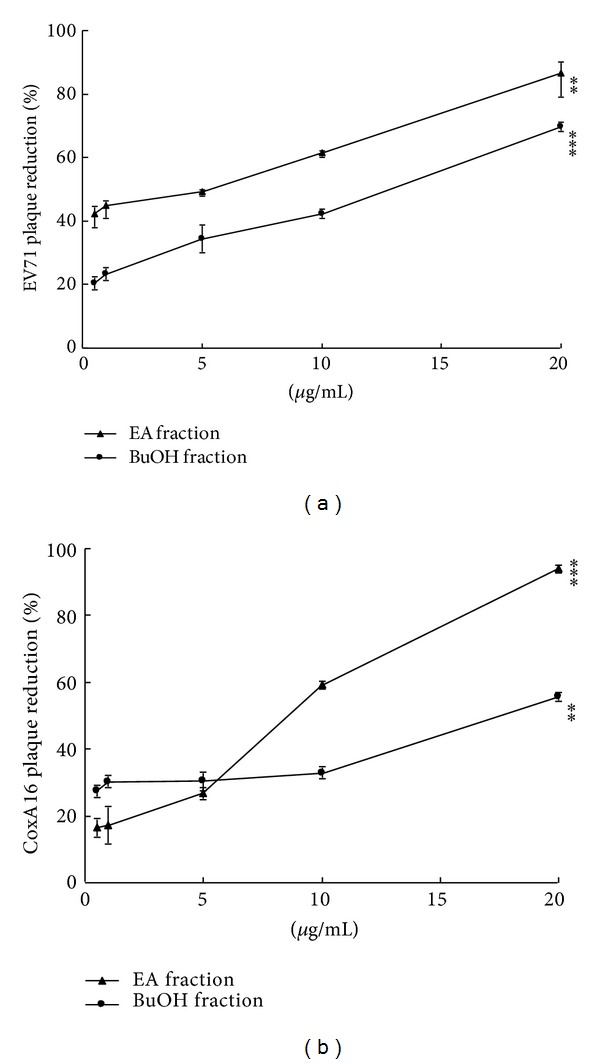
Plaque reduction by EA and BuOH fractions of KGS extract. Lyophilized KGS extract powder was dissolved in water, then fractionated by ethyl acetate (EA), followed by *n*-butanol (BuOH). Each fraction was plaque assayed for antiviral activity against EV71 (a) and CoxA16 (b).

To evaluate potential antiviral fractions, KGS extract was further fractionated sequentially with EA, BuOH, and water; lyophilized powder of these three fractions was subsequently used to examine cytotoxicity and apoptosis to RD cells and antiviral effects on virus yield and plaque reduction (Figures 3-4). MTT assay elicited CC_50_ values of KGS EA, BuOH, and H_2_O fractions above 400 *μ*g/mL (Table 1), indicating these as less cytotoxic to RD cells. Plaque reduction assay indicated EA and BuOH, but not H_2_O fraction, effectively inhibiting EV71 more than CoxA16 replication *in vitro* (Figures 4(a)-4(b), Table 1). Antienterovirus activity and selectivity of fractions in plaque reduction assay was ranked in the following order: EA (IC_50_ = 4.21 ~ 9.08 *μ*g/mL; SI = 45.14 ~ 97.35) > BuOH (IC_50_ = 11.88 ~ 18.23 *μ*g/mL; SI = 23.34 ~ 35.82) > H_2_O (IC_50_ = >100 *μ*g/mL) (Figures 4(a)-4(b), Table 1). Moreover, cell cycle analysis using flow cytometry indicated EV71 and CoxA16 infection causing significant increases of apoptosis (sub-G1 phase) in RD cells as well as EA and BuOH treatment markedly reducing the apoptosis from about 70% to near 20% (Supplemental Figure 1). Results verify EA fraction exhibiting significantly higher antiviral activity against EV71 and CoxA16 than BuOH fraction, containing potential antienterovirus components.

### 3.3. Eupafolin as Potent Antiviral Component in KGS EA Fraction against EV71 and CoxA16

**Figure 5:**
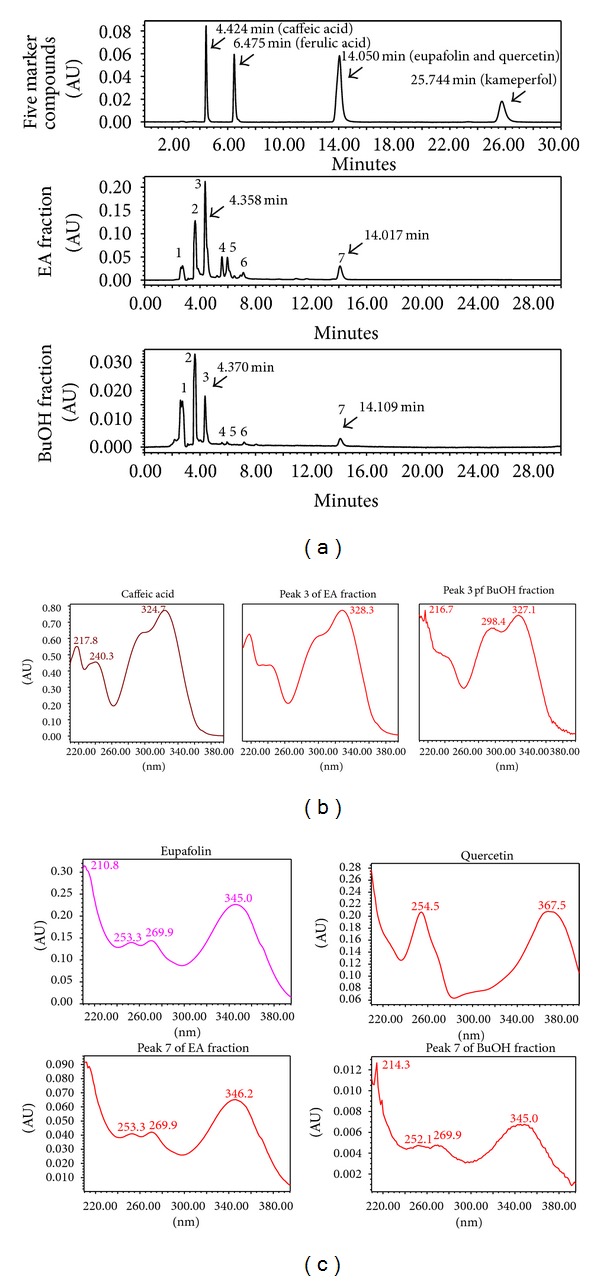
HPLC fingerprint profiles and UV/Vis absorption spectra of EA and BoOH fractions. Marker components (caffeic acid, ferulic acid, quercetin, eupafolin, and kaempferol), as well as both fractions of KGS extract, were analyzed by HPLC with C-18 reverse phase column, eluents detected at 345 nm with a 2996 PDA detector (a). Maximum absorption wavelengths of caffeic acid, quercetin, eupafolin, and chromatographic peaks 3 and 7 were measured by UV/Vis absorption spectra (200–360 nm) ((b), (c)).

**Figure 6:**
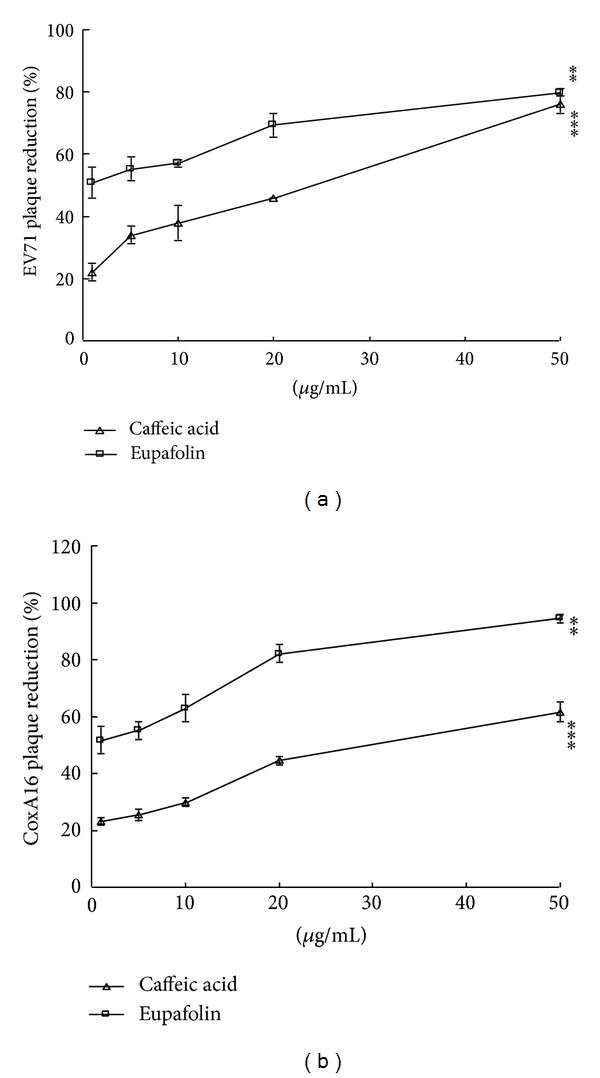
Plaque reduction by marker components: caffeic acid and eupafolin. Caffeic acid and eupafolin were analyzed for antiviral activity against EV71 (a) and CoxA16 (b), using plaque assay.

To examine fingerprint of KGS fractions, ferulic acid, quercetin, kaempferol, caffeic acid, and eupafulin served as standard marker components; EA and BuOH fractions were analyzed using HPLC with C-18 reverse phase column (Figure 5(a)). The retention time of HLPC chromatograph at 345 nm was at 4.424 min for caffeic acid, 6.475 min for ferulic acid, 14.050 min for eupafolin (overlapped with quercetin), and 25.744 min for kameperfol, respectively. HPLC chromatogram indicated retention times of Peak 3 and Peak 7 of EA and BuOH fractions as consistent with caffeic acid and eupafolin (or quercetin), respectively. UV absorption spectra (200–400 nm) indicated Peak 3 as the similar profile to caffeic acid and Peak 7 as the same profile of eupafolin, not quercetin (Figures 5(b)-5(c)). HPLC chromatogram and UV absorption spectra demonstrated EA and BuOH fractions consisting of caffeic acid and eupafolin. Based on the calibration curve of standard markers, amount of eupafolin was 3.10 ± 0.09 *μ*g/mg in EA fraction and 0.32 ± 0.01 *μ*g/mg in BuOH fraction, respectively. The amounts of caffeic acid in EA and BuOH fraction were 10.91 ± 0.34 *μ*g/mg and 0.80 ± 0.02 *μ*g/mg, respectively. Higher amounts of caffeic acid and eupafolin could account for better antienterovirus activity of EA versus BuOH fraction. Subsequently, antiviral activity of caffeic acid and eupafolin against EV71 and CoxA16 were rated by plaque reduction assay (Figure 6). Eupafolin had potent antiviral activity, with IC_50_ values of 0.44 *μ*g/mL (1.39 *μ*M) for EV71 and 1.66 *μ*g/mL (5.24 *μ*M) for CoxA16, showing excellent selectivity with SI values of 808.80 for EV71 and 214.38 for CoxA16 (Figure 6, Table 1). Meanwhile, IC_50_ values of caffeic acid were 23.87 *μ*g/mL for EV71 and 35.51 *μ*g/mL for CoxA16. Caffeic acid showed less antiviral activity than eupafolin, which indicated eupafolin playing a key role in anti-EV71 and CoxA16 actions of KGS EA fraction.

### 3.4. Virucidal Activity by Eupafolin

**Figure 7:**
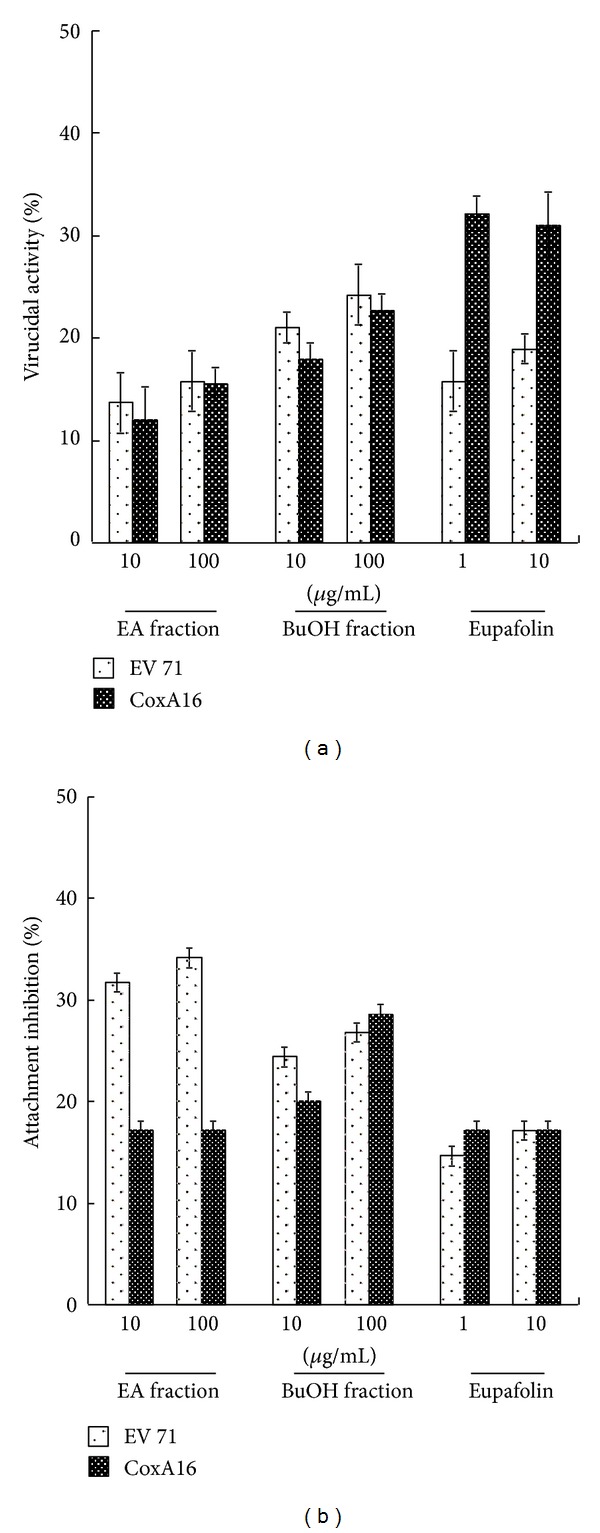
Virucidal activity and attachment inhibition of eupafolin, EA, and BuOH fractions. In virucidal assay (a), eupafolin or each fraction was mixed with EV71 or CoxA16 (10^6^ pfu), then incubated at 4°C for 1 h. Residual infectivity was performed by plaque assay with 1000-fold dilution of virus/compound mixture. In the attachment assay (b), EV71 or CoxA16 (50 pfu) was mixed with EA, BuOH fraction, or eupafolin, then immediately added onto RD cell monolayer for 1 h at 4°C. After washing twice with PBS, monolayer was overlaid with 2 mL of agarose medium for 2 days at 37°C in CO_2_ incubator. Attachment inhibition was calculated as residual plaques after crystal violet staining.

**Figure 8:**
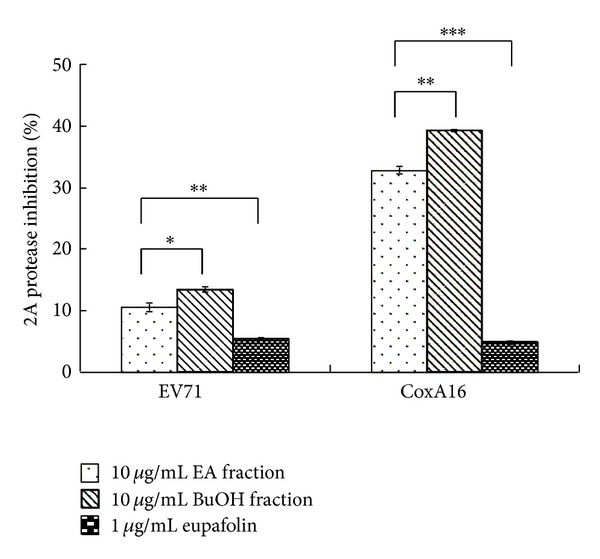
Inhibitory effect of eupafolin, EA, and BuOH fractions on viral 2A protease activity in cell-based FRET assay. HeLa-G2AwtR cells expressed 2A-cleavage motif in FRET probes were infected with EV71 or CVA16 at a MOI of 1, coexistent with treatment of eupafolin, EA, and BuOH fractions. Cells harvested 48 postinfection were subjected to measurement by a fluorescent-plate reader with excitation wavelength at 390/20 nm and emission wavelength at 510/10 nm (for GFP^2^) or 590/14 nm (for DsRed2). Inhibitory activity was calculated as FRET ratio, that is, intensity of emission at 590/14 nm divided by that at 510/10 nm. *P value < 0.05; **P value < 0.01; ***P value < 0.001 by Scheffe's test.

To evaluate possible direct-acting antiviral mechanism, eupafolin, EA, and BuOH fractions were tested for virucidal, attachment, and viral protease inhibition. In virucidal activity assay (Figure 7(a)), EA and BuOH fractions at 100 *μ*g/mL showed low virucidal activity for EV71 and CoxA16 (reduction less than 25%). Eupafolin at 1 or 10 *μ*g/mL reduced CoxA16 infectivity by 30%, but slightly affected EV71 infectivity (lower than 20%). In attachment inhibition assay (Figure 7(b)), only EA fraction at 10 or 100 *μ*g/mL inhibited above 30% of EV71 binding to RD cells. In cell-based viral 2A protease activity assay (Figure 8), EA and BuOH fractions at 10 *μ*g/mL inhibited CoxA16 2A protease activity by over 30%. Still, eupafolin had no significant inhibitory effect on EV71 and CoxA16 activity. Results show difference in direct-acting antiviral actions by KGS EA and BuOH fractions. Eupafolin had moderate virucidal effect against both enterovirus types.

### 3.5. Inhibition of Virus-Induced Proinflammatory Cytokines by Eupafolin

**Figure 9:**
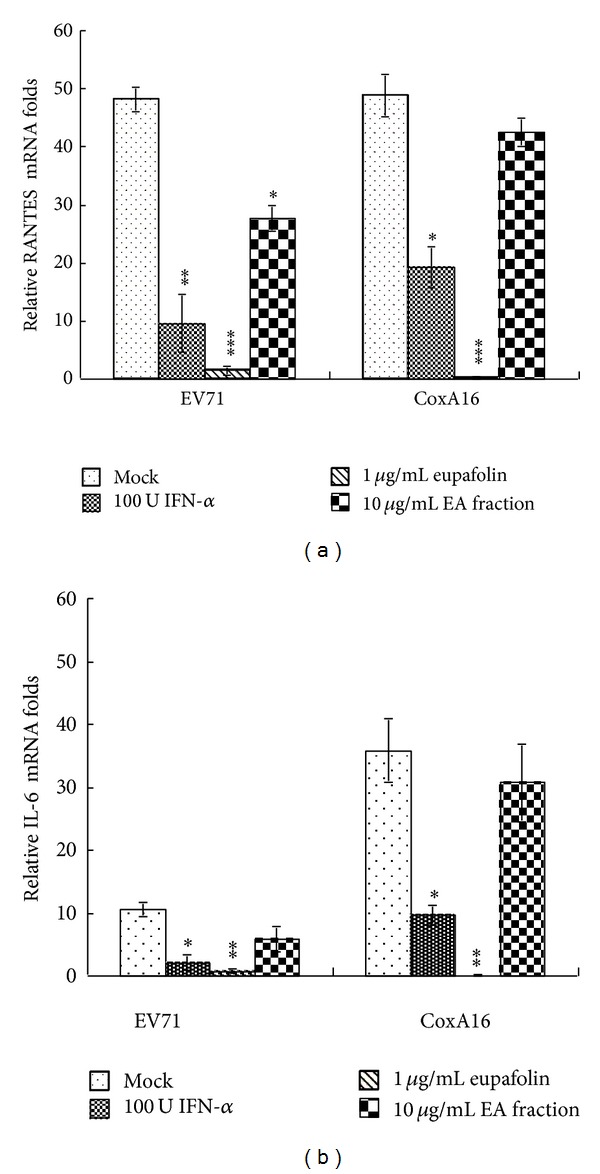
Expression levels of proinflammatory genes in infected RD cells treated with/without eupafolin and EA fraction. IL-6 (a) and RANTES (b) mRNA in RD cells 8 h postinfection and treatment were extracted and measured with real-time RT-PCR. Relative mRNA expression levels were normalized by housekeeping gene GAPDH. *P value < 0.05; **P value < 0.01; ***P value < 0.001 by Scheffe's test.

**Figure 10:**
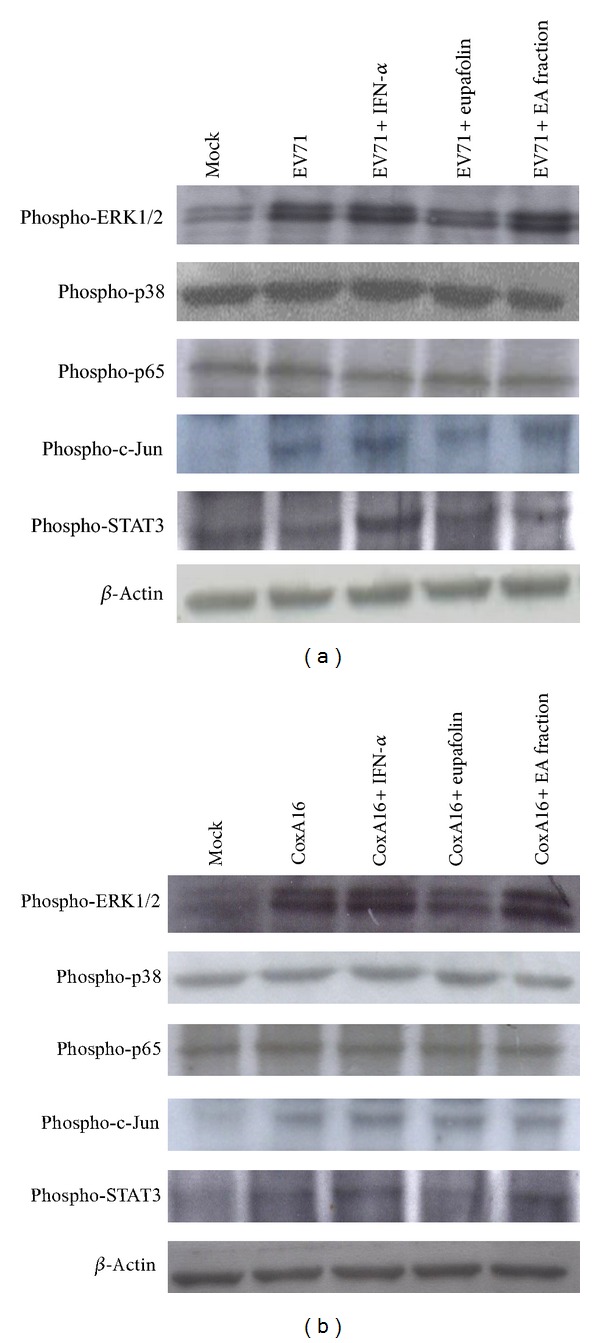
Phosphorylation levels of ERK1/2, p38 MAPK, p65 (NF-*κ*B), c-Jun, and STAT3 in infected RD cells treated with(out) eupafolin and EA fraction. EV71 (a) or CoxA16 (b) infected RD cells were harvested 9 h posttreatment, and lysates resolved on 10% SDS-PAGE and transferred onto nitrocellulose paper. Blot was probed with specific mAbs, developed with alkaline phosphatase-conjugated secondary antibody and enhanced chemiluminescence substrates. Lane 1, mock cells; Lane 2, infected cells; Lane 3, infected cells treated with IFN-*α*; Lane 4, infected cells treated with eupafolin; Lane 5, infected cells treated with EA fraction.

To examine effect of eupafolin on virus-induced proinflammatory cytokine expression further, relative IL-6 and RANTES mRNA levels in virus-infected cells treated with/without eupafolin, IFN-*α*, or EA fraction were derived by quantitative real-time PCR (Figure 9). Eupafolin at 1 *μ*g/mL decreased virus-induced IL-6 and RANTES expression by more than 10-fold, suppressing proinflammatory cytokines induced by EV71 and CoxA16 better than KGS EA fraction (10 *μ*g/mL) and IFN-*α* (100 U/mL). Phosphorylation levels of cytokine induction-related proteins p38 MAPK, ERK1/2, NF-*κ*B (p65), c-Jun, and STAT3 in virus-infected cells treated with or without eupafolin, IFN-*α*, or EA fraction were subsequently analyzed by Western blotting via phosphorylation site-specific antibodies (Figure 10). EV71 and CoxA16 infections raised phosphorylation of ERK1/2, c-Jun, and STAT3, but not p38 MAPK and NF-*κ*B p65, 9 h postinfection. Treatment with eupafolin attenuated activation of ERK1/2, c-Jun, and STAT3 in EV71- and CoxA16-infected cells. Data confirm eupafolin suppressing IL-6 and RANTES expressions and reducing ERK1/2, AP-1, and STAT3 mediated signaling induced by EV71 and CoxA16, suggesting anti-inflammatory effect of eupafolin as involved in antiviral activity against EV71 and CoxA16.

## 4. Discussion

KGS extract, EA, and BuOH fractions, as well as its components eupafolin and caffeic acid, processed low cytotoxicity (Figure 1, Table 1). KGS EA fraction had an antienterovirus activity with IC_50_ values less 10 *μ*g/mL, more effectively inhibiting replication of EV71 and CoxA16 *in vitro* than KGS extract and BuOH fraction (Figures 2–4, Table 1). EA fraction contained antiviral components: eupafolin (3.10 ± 0.09 *μ*g/mg) with IC_50_ values of 1.39 *μ*M for EV71 and 5.24 *μ*M for CoxA16, as well as caffeic acid (10.91 ± 0.31 *μ*g/mg) with IC_50_ values of 132.50 *μ*M for EV71 and 197.11 *μ*M for CoxA16 (Figures 5-6, Table 1). Our prior study proved quercetin an active antienterovirus component of* K. gracilis* leaf extract, showing IC_50_ values above 117 *μ*M for EV71 and 176 *μ*M for CoxA16 [14]. Eupafolin was thus suggested as crucially active antienterovirus component in* K. gracilis*, at the same time showing similar anti-EV71 efficacy and selectivity with identified potential anti-EV71 compounds of natural products like allophycocyanin, aloe-emodin, gallic acid, chrysosplenetin, and penduletin [17–20].

KGS extract reduced by more than 90% both cytopathicity and virus yield 36 h postinfection (Figure 2), implying antiviral activity linked with direct and indirect antiviral actions: for example, virucidal activity, attachment blocking, targeting viral enzymes, and host factors, inducing host antiviral responses. Our prior study [14] demonstrated *K. gracilis* leaf extract inhibiting viral 2A protease activity, reducing virus-induced apoptosis, as well as suppressing IL-6 and RANTES upregulation by EV71 and CoxA16. This study averred both KGS fractions plus eupafolin exhibiting low virucidal activity and slightly blocking virus attachment (Figures 7(a)-7(b)). Both KGS fractions inhibited CoxA16 2A proteases of CoxA16, but eupafolin failed to inhibit viral 2A proteases (Figure 8). Eupafolin specifically inhibited upregulation of IL-6 and RANTES gene expressions induced by EV71 or CVA16 infection (Figure 9), which correlated with reduction of virus-induced ERK1/2, c-Jun, and STAT3 mediated signaling (Figure 10). Both KGS fractions exhibited multiple inhibitory actions against EV71 and CoxA16, relating to decrease of viral infectivity, attachment, and protease enzymatic activity *in vitro*. Aside from virucidal activity and attachment inhibition, eupafolin significantly inhibited production of IL-6 and RANTES in enterovirus infection. EV71-infected patients' elevated levels of IL-1*β*, IL-6, and TNF-*α* in CSF strongly correlate with clinical severity [21, 22]. In addition, EV71 infection causes the upregulation of COX-2 and PGE(2) via activation of ERK1/2 and AP-1 signaling pathways [23]. Eupafolin significantly inhibited activation of ERK1/2, c-Jun, and STAT3 in both virus-infected cells, which correlates with suppressing upregulation of IL-6 and RANTES by eupafolin treatment. It thus processed potent antiviral and antiproinflammatory activities, displaying therapeutic potential against EV71 and CoxA16 infection. Combination of effective compounds of* K. gracilis*, including eupafolin, quercetin, and caffeic acid, could provide an alternative approach against enteroviral infection.

In sum, KGS extract contains potent antienteroviral components; fractionation augments antienteroviral effect. Eupafolin, a crucial antiviral component of KGS EA fraction, shows high selective index for EV71 and CoxA16 by greater than a 30-fold increase. Eupafolin is the potential enteroviral agent with anti-inflammatory activities via suppressing virus-induced activation of ERK1/2, AP-1, and STAT3-mediated signaling pathways.

## Supplementary Material

To examine the in vivo anti-EV efficacy of KGS extract, the sucking mice were intraperitoneally infected with EV71, and then simultaneously treated with KGS extract. The viral load in the intestinal sample indicated KGS extract inhibiting EV71 replication in vivo (Supplemental Table 1). In addition, EA and BuOH fractions of KGS extract were evaluated the reduction ability on viral cytopathicity with PI staining by flow cytometry analysis (Supplemental Figure 1). EA and BuOH fractions significantly inhibited the viral cytopathicity, as well as reducing apoptosis of virus-infected cells.

## Figures and Tables

**Table 1:** Cytotoxicity and antienteroviral activity of KGS extract, fractions, and marker components.

		EV71		COXA16	
	CC_50_ (*μ*g/mL) to RD cells	IC_50_ (*μ*g/mL)	SI	IC_50_ (*μ*g/mL)	SI
KGS extract	1622.30	75.18	21.58	81.41	19.93
EA fraction	409.83	4.21	97.35	9.08	45.14
BuOH fraction	425.53	11.88	35.82	18.23	23.34
H_2_O fraction	>500	>100		>100	
Eupafolin	355.87	0.44	808.80	1.66	214.38
Caffeic acid	274.72	23.87	11.51	35.51	7.74
