# Supplementary material for: Eupafolin and Ethyl Acetate Fraction of Kalanchoe gracilis Stem Extract Show Potent Antiviral Activities against Enterovirus 71 and Coxsackievirus A16
Source: Evid Based Complement Alternat Med. 2013 Sep 2;2013:591354. doi: 10.1155/2013/591354 (PMC3775429; doi:10.1155/2013/591354)
Supplement: Supplementary file 1 — To examine the in vivo anti-EV efficacy of KGS extract, the sucking mice were intraperitoneally infected with EV71, and then simultaneously treated with KGS extract. The viral load in the intestinal sample indicated KGS extract inhibiting EV71 replication in vivo (Supplemental Table 1). In addition, EA and BuOH fractions of KGS extract were evaluated the reduction ability on viral cytopathicity with PI staining by flow cytometry analysis (Supplemental Figure 1). EA and BuOH fractions significantly inhibited the viral cytopathicity, as well as reducing apoptosis of virus-infected cells. [file 591354.f1.docx]

Supplemental Table 1.EV71 virus loads in pooled intestines of each group 2, 4, 6, and 8 days post treatment with KGS

| Group | Day 2 (pfu/ml) | Day 4 (pfu/ml) | Day 6 (pfu/ml) | Day 8 (pfu/ml) |
| --- | --- | --- | --- | --- |
| Negative control | ND | ND | ND | ND |
| Positive control | 3.7×10^5^ | 3.1×10^3^ | 2.8×10^3^ | ND |
| 5 mg/Kg KGS | 2.0×10^5^ | ND | ND | ND |

ND: not detectable


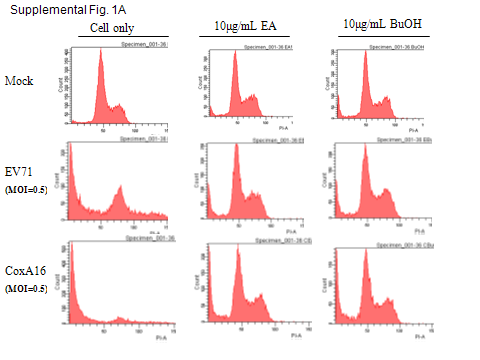


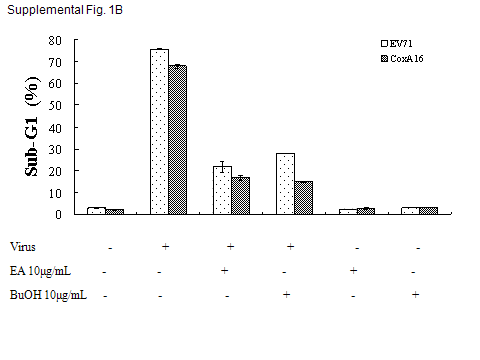


**Supplemental Figure 1. Cell cycle analysis (A) and apoptosis rate (B) of EV71 or CoxA16 virus-infected cells 36 h post treatment with(out) EA and BuOH fraction.**
